# Supplementary material for: Addressing erroneous scale assumptions in microbe and gene set enrichment analysis
Source: PLoS Comput Biol. 2023 Nov 20;19(11):e1011659. doi: 10.1371/journal.pcbi.1011659 (PMC10695402; doi:10.1371/journal.pcbi.1011659)
Supplement: S1 Text — (PDF) [file pcbi.1011659.s001.pdf]

Supplement to Addressing Erroneous Scale Assumptions in Microbe and Gene Set Enrichment Analysis

Kyle C. McGovern[1], Michelle Pistner Nixon[2], and Justin D. Silverman[1,2,3,4,5]

[1]Program in Bioinformatics and Genomics, Pennsylvania State University

[2]College of Information Sciences and Technology, Pennsylvania State University

[3]Departments of Medicine and Statistics, Pennsylvania State University

[4]Institute for Computational and Data Science, Pennsylvania State University

[5]Corresponding Author: JustinSilverman@psu.edu

A Inter-Entity Correlations

In the main text we focus on GSEA-LFC with respect to entity-label permutations which implicitly assumes that the entities are statistically independent: an assumption that can be violated in practice leading to false positives [1–3]. Here we extend the results of the main text to three DSA methods that account for inter-entity correlations. The first two methods are GSEA-LFC-S and GSEA-CLR-S. These methods are GSEA-LFC and GSEA-CLR respectively but with the suffix *-S* indicating the use of sample-label (rather than entity-label) permutations which many argue maintain the correlation structure of the data [4, 5]. We also consider the CAMERA [2] method which looks for a difference in the mean LFC between entities in and out of the set using a t-test while including an inter-entity correlation correction factor.

We initially explored the sensitivity of GSEA-LFC-S to errors in scale assumptions using the Aran et al. thyroid tissue dataset [6] that we reanalyzed in the Section *LFC Sensitivity Analysis and Testing of Real Data* in the main text. Remarkably, in changing from using entity-label permutations (as used by Aran et al. [6]), to sample-label permutations, GSEA-LFC-S returned no significant hits. This suggests that the results reported in Aran et al. could be false positives secondary to an erroneous assumption that the entities were independent (i.e., the gene expression was not correlated). While these results highlight the importance of considering inter-entity correlations, it also implied that this dataset was likely a poor test-case for studying the role of scale-limitations when accounting for inter-entity correlations. As a result, here we focus on a different dataset consisting of 184 samples of either healthy or cancer breast tissue and a much larger list of 4622 genes sets (see Sections A.1 and A.2).

Performing a sensitivity analysis of this breast cancer dataset also required generalizing LFC Sensitivity Analysis beyond the two-step LFC-based estimators mentioned in the main text. We adopt a more general Scale Sensitivity Analysis as discussed in Nixon et al. [7]. For LFC Sensitivity Analysis we perform sensitivity analysis of the target estimand (ϕ) as a function of error $\epsilon^{\perp}$ in the estimated LFCs ($\hat{\theta}$): $\phi_S = u(\hat{\theta} + \mathbf{1}\epsilon^{\perp})$. The simplicity of this approach relied in part on the fact that the estimate of the log-fold-change in scales between conditions (i.e., $\hat{\theta}^{\perp}$) did not change under entity-label permutations. However, for GSEA-LFC-S the estimate $\hat{\theta}^{\perp}$, will change depending on which samples are assigned to the case and control groups after sample-label permutation. Instead, we perform Scale Sensitivity Analysis by evaluating the error in the applied estimand after adding sample-specific scale error $\alpha_n^{\perp}$ to each sample on the log scale. As in LFC Sensitivity Analysis, we assume that there is limited compositional error such that $W^{\parallel} \approx \hat{W}^{\parallel}$. Similar to LFC Sensitivity Analysis we may then express the estimated scaled system on the log scale for entity d and sample n as

$$\begin{aligned}
\log W_{dn} &= \log W_{dn}^{\parallel} + \log W_n^{\perp} \\
&\approx \log \hat{W}_{dn}^{\parallel} + \left(\log \hat{W}_n^{\perp} + \alpha_n^{\perp}\right) \\
&\approx \log \hat{W}_{dn} + \alpha_n^{\perp}.
\end{aligned}$$

Scale Sensitivity Analysis is a sensitivity analysis comparing the applied estimand $\hat{\phi}$ to the target estimand ϕ when the applied estimand has errors $\alpha_1^{\perp}, \ldots, \alpha_n^{\perp}$: $\phi_S = u\left(f\left(\log \hat{W}_{\cdot,1} + \alpha_1, \ldots, \log \hat{W}_{\cdot,n} + \alpha_n^{\perp}\right)\right)$ where the function f returns LFCs $\hat{\theta}$ for GSEA-LFC-S and GSEA-CLR-S. For CAMERA the matrix $\hat{W}$ is used directly, without first summarizing the data as LFCs, so f can be thought of as the identity function.

We conducted two Scale Sensitivity Analyses whose results are visualized in Fig A. In the first sensitivity analysis (Fig A part a) we simulate error $\alpha_n^{\perp}$ as some constant $\delta^{\perp}$:

$$\alpha_n^{\perp} = \begin{cases} \delta^{\perp}, & \text{if } x_n = 1 \\ 0, & \text{if } x_n = 0. \end{cases}$$

In this particular sensitivity analysis, error ($\delta^{\perp}$) is constant across samples in the diseased condition and 0 for samples in the healthy experimental condition. As a result this analysis has a similar interpretation to error in $\hat{\theta}^{\perp}$ (i.e., $\theta^{\perp} = \hat{\theta}^{\perp} + \epsilon^{\perp}$) because

$$\begin{aligned}
\theta^{\perp} &= \underset{n:x_n=1}{\text{mean}}(\log \hat{W}_n^{\perp} + \delta^{\perp}) - \underset{n:x_n=0}{\text{mean}}(\log \hat{W}_n^{\perp} + 0) \\
&= \underset{n:x_n=1}{\text{mean}}(\log \hat{W}_n^{\perp}) - \underset{n:x_n=0}{\text{mean}}(\log \hat{W}_n^{\perp}) + \delta^{\perp} \\
&= \hat{\theta}^{\perp} + \delta^{\perp}.
\end{aligned}$$

It should be noted however that Scale Sensitivity Analysis is still required for this analysis as the diseased

and healthy condition labels (x_n) change with sample-label shuffling, and thus the estimated means change as well.

In the second Scale Sensitivity Analysis shown in Fig A part b we simulated distinct, independent error in each $\log \hat{W}_n^{\perp}$ for some constant $\gamma^{\perp}$ from the uniform distribution (denoted as U):

$$\alpha_n^{\perp} \sim U[-\gamma^{\perp}, \gamma^{\perp}].$$

Unlike the first Scale Sensitivity Analysis, here an element of randomness has been introduced in sampling from the uniform distribution and thus we performed this Scale Sensitivity Analysis for 50 independent samples of $\alpha_1^{\perp}, ..., \alpha_N^{\perp}$. We discuss the results of these two Scale Sensitivity Analyses in the remainder of this section.

Like the LFC Sensitivity Analyses in the main text, here we use the Positive Predictive Value (PPV) as a measure of sensitivity for different values of $\alpha_1^{\perp}, \ldots, \alpha_N^{\perp}$ tested. When there is no sensitivity to scale errors we expect the PPV to be 100% across all scale assumption errors tested. Our results show that GSEA-CLR-S is completely insensitive (with a PPV of 100%) to all errors in scale assumptions (Fig A, blue diamonds and blue box plots). GSEA-CLR-S was still able to identify 595 putative hits despite this scale insensitivity. We found that GSEA-LFC-S is sensitive to errors $\delta^{\perp}$ in $\hat{\theta}^{\perp}$ (Fig A part a, orange X symbols) with the PPV reaching as low as 54%. GSEA-LFC-S is however not nearly as sensitive to random errors in $\hat{W}^{\perp}$ (Fig A part b, orange boxplots) with the PPV only dropping to 96%. We found that CAMERA is completely insensitive to errors in $\hat{\theta}^{\perp}$ (Fig A part a, blue diamonds), but shows a high degree of sensitivity to errors in $\hat{W}^{\perp}$ (Fig A part b, blue boxplots). For errors in $\hat{W}^{\perp}$ sampled uniformly when $\gamma^{\perp} = 0.05$, CAMERA's PPV dropped to 94% and to as low as 53% when $\gamma^{\perp} = 0.25$.

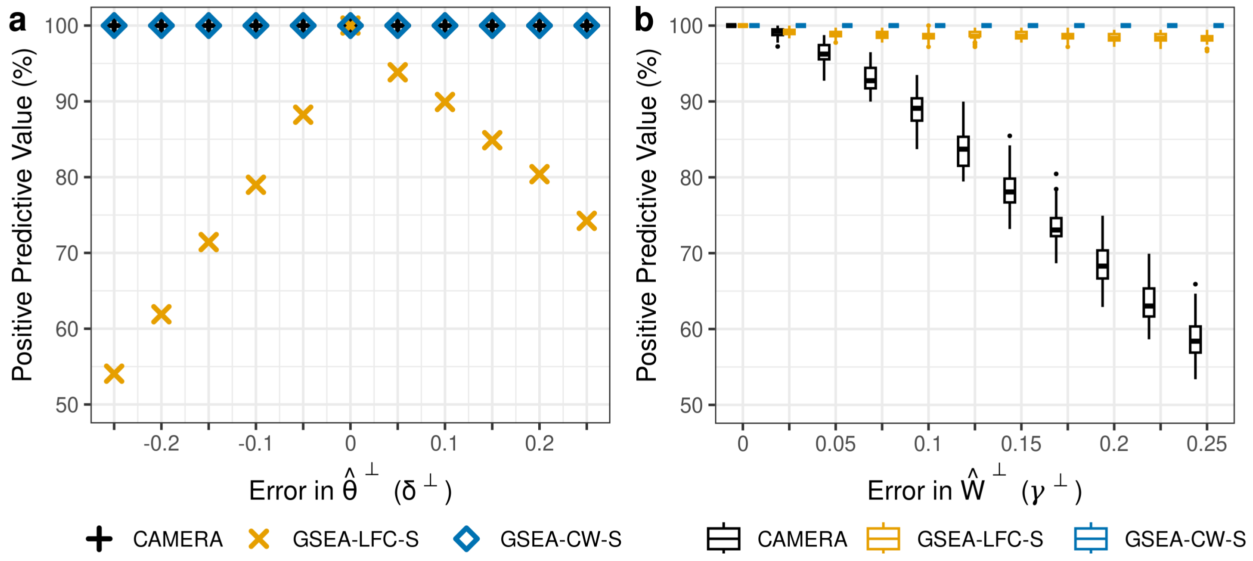

Figure A: Common methods that account for inter-entity correlations such as CAMERA and GSEA-LFC-S are scale reliant while GSEA-CW-S is scale invariant. The figure represents the output of two Scale Sensitivity Analyses performed on a study comparing gene expression in healthy ($n = 92$) versus tumor ($n = 92$) breast tissue using a database of 4622 gene sets (see Sections A.1 and A.2 for further details). (a) The result of a scale sensitivity analysis where scale assumption error $\alpha_n^{\perp}$ is $\delta^{\perp}$ if $x_n = 1$ (i.e., tumor tissue) and 0 if $x_n = 0$ (i.e., healthy tissue). Error $\delta^{\perp}$ can be thought of as error in the scale assumption $\hat{\theta}^{\perp}$ because it is constant: $\theta^{\perp} = \hat{\theta}^{\perp} + \delta^{\perp}$. (b) Box plots summarizing the PPVs observed from 50 simulations of scale assumption α where each $\alpha_1^{\perp}, \dots, \alpha_N^{\perp}$ is sampled from the uniform distribution with upper and lower bounds $\gamma^{\perp}$, (e.g., $U(-\gamma^{\perp}, \gamma^{\perp})$). This error can be interpreted as error in $W^{\perp}$: for each element n of the N length vector $W^{\perp}$: $W_n^{\perp} = \hat{W}_n^{\perp} + \alpha_n^{\perp}$ where $\alpha_n^{\perp} \sim U(-\gamma^{\perp}, \gamma^{\perp})$.

We also found that the severity of the scale sensitivity for GSEA-LFC-S is gene set dependent and that this may have implications for actual research. The Cell Cycle and Mismatch Repair pathways have both been identified in the literature as enriched in breast cancer tissue [8], yet here we find that only the former pathway is enriched in breast cancer tissue across a wide range of tested values of $\delta^{\perp}$ while the latter is enriched only over a very narrow range of values of $\delta^{\perp}$ (Fig B).

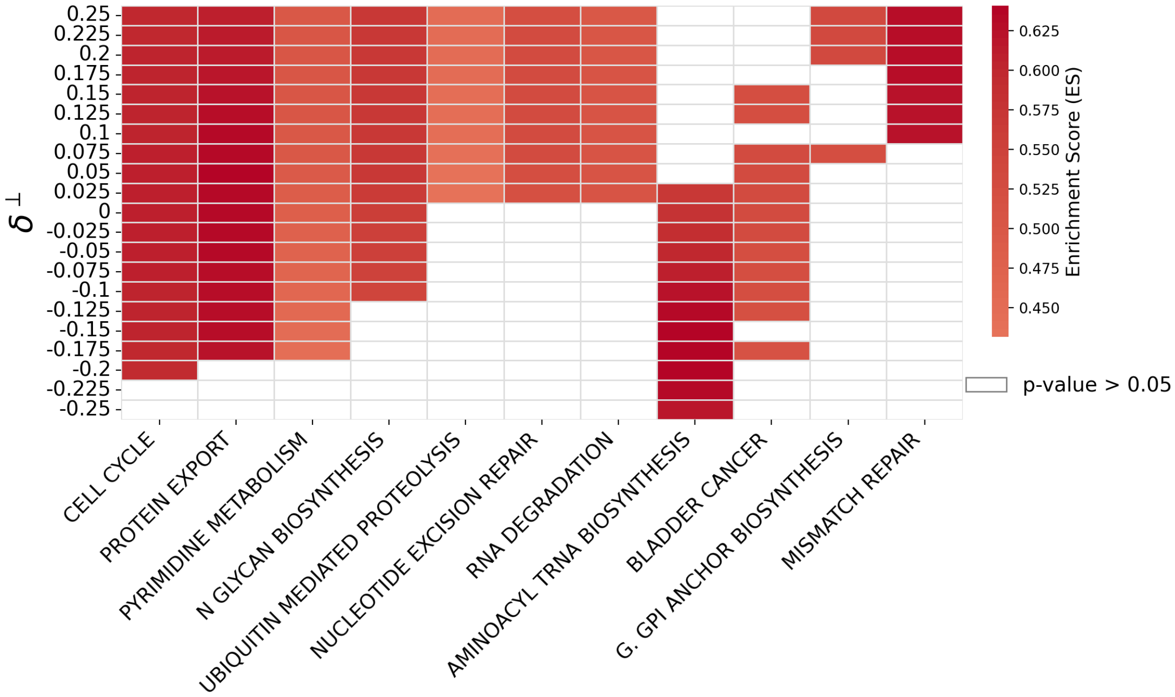

Figure B: Gene sets identified by GSEA-LFC-S have different degrees of sensitivity to errors in scale assumptions. Gene set specific results are presented for the Scale Sensitivity Analysis presented in Fig A part a. $\delta^{\perp}$ may be interpreted as error in the assumed log-fold-change in scales $\hat{\theta}^{\perp}$: $\theta^{\perp} = \hat{\theta}^{\perp} + \delta^{\perp}$.

Overall, our results are in line with the central findings of the main text: both GSEA-LFC-S and CAMERA are sensitive to errors in scale assumptions, albeit with the added complexity that the sensitivity depends on whether there is random scale error for each independent sample (e.g., $\alpha_n^{\perp} \sim U[-\gamma^{\perp}, \gamma^{\perp}]$) or constant scale error that differs only between experimental conditions (e.g., $\delta^{\perp}$). The observation that GSEA-CLR-S is scale invariant in both of our sensitivity studies similarly aligns with our findings presented in Section F that GSEA-CW (and by extension GSEA-CLR-S) is scale invariant.

A.1 Methods for Preprocessing of Breast Tissue RNA-seq Data

The pre-processed read count matrices were downloaded from NCBI's GEO under accession numbers GSE86354 (for healthy tissue) and GSE62944 (for tumor tissue) [6, 9, 10]. These datasets contained samples from a variety of tissues, therefore samples were filtered until only 1,119 breast tumor tissue samples and 92 healthy breast tissue samples remained. To prevent the Scale Sensitivity Analyses from being impacted by class imbalance, we sub-sampled the number of tumor samples to 92 to match the number of samples in the healthy condition then merged the read count matrices together by gene name. After sample filtering, many genes contained exclusively zero counts; thus, to ensure adequate signal in both conditions we only included

genes that had non-zero counts in at least 3 replicates in both the healthy and tumor conditions, resulting in a filtered set of 22,280 genes.

A.2 Methods for Scale Sensitivity Analyses

Both the Scale Sensitivity Analyses were performed using the MSigDB C2 (version 7.4.0) curated list of gene sets [4, 11]. Based on the default parameters of the GSEA software package [4], only gene sets that containing between 15 and 500 genes were retained in our analysis resulting in a set of 4622 candidate gene sets. A pseudo-count of 0.1 was added to the read count matrix prior to CLR transformation for numerical stability. In the first sensitivity analysis, error α_n was added to the columns of the CLR transformed count matrix $\hat{W}^{CLR}$ where $\alpha_n = \delta^{\perp}$ if $x_n = 1$ (i.e., tumor tissue samples) and $\alpha_n = 0$ if $x_n = 0$ (i.e., healthy tissue samples). In the second Scale Sensitivity Analysis presented, errors sampled from the uniform distribution as $\alpha_n^{\perp} \sim U[-\gamma^{\perp}, \gamma^{\perp}]$ for a constant $\gamma^{\perp}$ were added to the matrix $\hat{W}^{CLR}$. For GSEA-LFC, LFCs $\hat{\theta}$ were calculated from the matrix $\hat{W}^{CLR}$ using Eq (5) in the main text. For GSEA-LFC-S, LFCs were also calculated using Eq (5) in the main text, but LFCs were also recalculated after each sample label permutation to form the null distribution. For CAMERA, rather than calculating LFCs, inter-entity correlations were first calculated using the matrix $\hat{W}^{CLR}$ as input to the *interGeneCorrelation* function in the Limma R package version 5.50.3 [12]. Then the estimated inter-entity correlations and matrix $\hat{W}^{CLR}$ were used as input to the *cameraPR* function which returned p-values for enrichment or depletion. All p-values were calculated using a significance threshold of $p < 0.05$ and 5000 sample-label permutations.

B Gene Set Enrichment Analysis

In this section we provide a thorough definition of Gene Set Enrichment Analysis (GSEA) [4]. Subramanian et al. [4] define the GSEA test statistic as the Enrichment Score (ES) which is the supremum (i.e., maximum distance from 0) of the difference of two running sums ($P_{hit} - P_{miss}$) [4]. For an entity set S of size D_S, for each entity $d \in [1, \ldots, D]$ in the list ranked by a ranking stat R_d, P_{hit} and P_{miss} are calculated as

$$P_{hit}(S, d) = \sum_{\substack{j \leq d \\ j \in S}} \frac{|R_j|}{N_R}, \text{ where } N_R = \sum_{j \in S} |R_j|$$

$$P_{miss}(S, d) = \sum_{\substack{j \leq d \\ j \notin S}} \frac{1}{D - D_S}.$$

Letting $R_d = \theta_d$ (where θ_d represents the LFC for entity d) we obtain GSEA-LFC(-S). In contrast letting $R_d = f(l_d(\theta))$, where $l_d(\theta)$ denotes a log-contrast of the vector θ and f represents an arbitrary real-valued function, results in CSEA-CW(-S) (see Section F for more details).

A p-value is determined for GSEA using a null distribution created by permuting either the entity labels

or sample labels. For entity label permutations, the entities in the entity set S are randomly reassigned. For sample label permutations, the condition labels (i.e., $x_1, \ldots, x_n$) are permuted, randomly changing whether each sample belongs to the case or control condition. The p-value is not calculated using the entire null distribution; only those permuted test statistics with the same sign (i.e., positive or negative) as the observed test statistic are used to calculate the p-value. The p-value is calculated as the proportion of the permuted test statistics (with the same sign) that are absolutely greater than or equal to the observed test statistic.

C A Larger Set of LFC Sensitivity Simulations for GSEA-LFC

In the Section *The GSEA-LFC Target Estimand is Typically Scale Reliant* and Fig 1C in the main text we presented a single, small simulation experiment to demonstrate that GSEA-LFC is scale reliant and that Positive Predictive Values (PPVs) can be as low as 9%. Here we describe a larger simulation study to explore how the sensitivity of GSEA-LFC to errors in scale assumptions depends on a range of factors such as the total number of entities (D), the size of the entity sets (D_S), and the distribution of the simulated LFCs (P).

Based on prior work (e.g., [2]), we hypothesized that highly skewed LFC distributions would result in lower PPVs. We chose the distribution of LFCs (P) to be either a normal distribution, uniform distribution, or left- or right-skewed distribution (Fig C part a). The left- and right-skewed distributions were created by randomly simulating 10% of the LFC from a uniform distribution on the interval $[-1, 0]$ and $[0, 1]$, respectively, and setting the remaining 90% of LFC to 0.01 or -0.01, respectively (Fig C part a). We chose D to be 500, 10,000, or 20,000 and D_S to be 15, 50, or 150. For each possible combination of P, D, and D_S we simulated 10,000 entity sets and performed our PPV-based LFC Sensitivity Analysis over a range of values $\epsilon^{\perp} \in (-2, 2)$ using a significance cutoff of $p < 0.05$ and 5000 entity-label permutations. As hypothesized, the asymmetric LFC distributions resulted in substantially lower PPVs than the other distributions while the impact of changing D_S or D was minimal comparatively (Fig C part b). These effects can be dramatic: when D_S=150 with an asymmetric distribution of LFCs we observed a PPV of only 0.2% with a value of $\epsilon^{\perp}$ as small as ± 0.6.

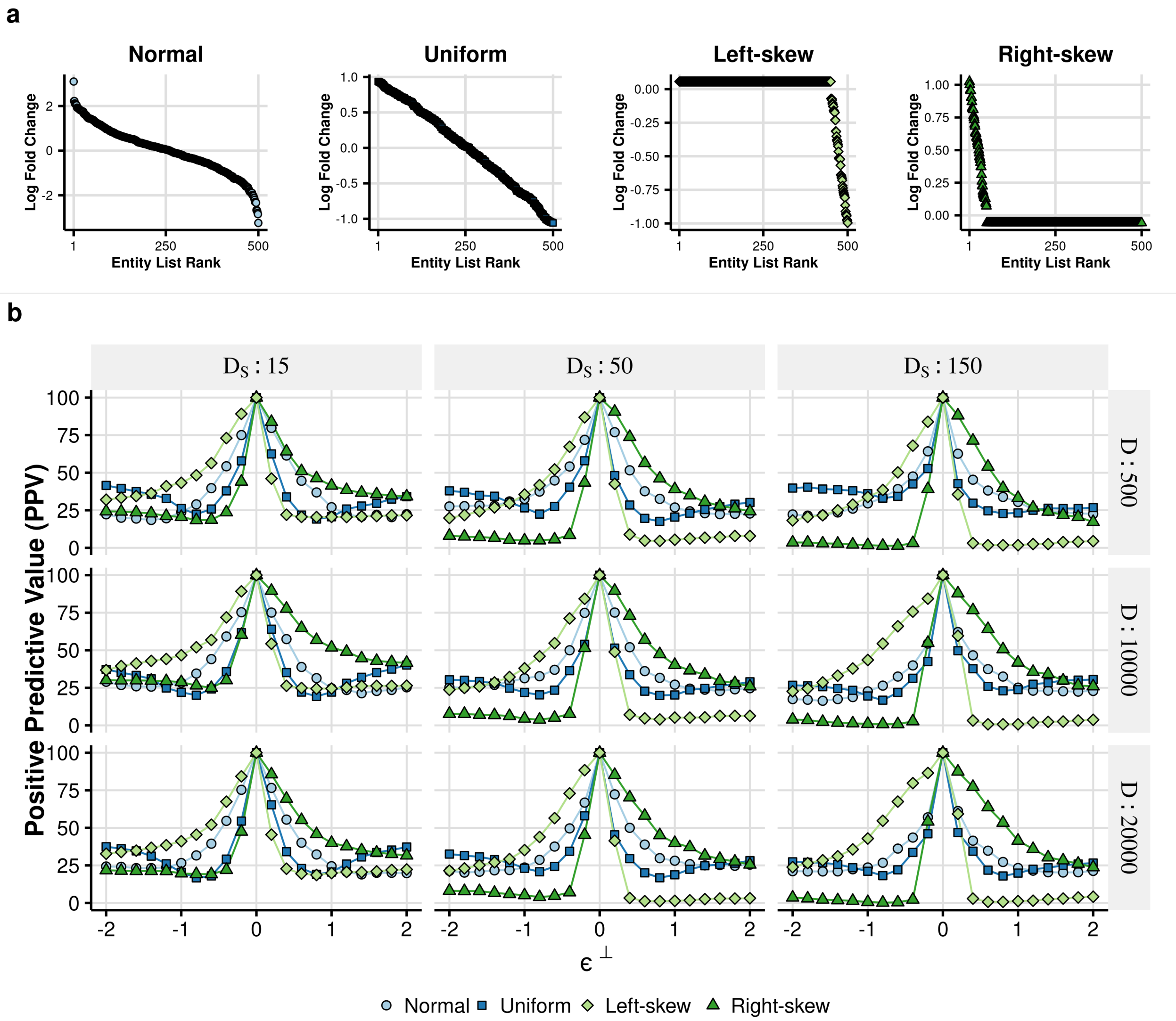

Figure C: Results of Larger Simulation Study of LFC Sensitivity in GSEA-LFC. (a) Example plots visualizing the four distribution types (P) considered. (b) The results of the LFC Sensitivity Analysis are plotted in a grid. The columns of the grid represent different entity set sizes (D_S) and the rows represent different total numbers of entities simulated (D). The PPV is calculated as the proportion of entity sets identified as significant ($p < 0.05$) by GSEA-LFC at the assumed $\hat{\theta}^{\perp}$ (i.e., when $\epsilon^{\perp} = 0$) that were also identified when the true log-fold-change in scales is instead $\theta^{\perp} = \hat{\theta}^{\perp} + \mathbf{1}\epsilon^{\perp}$.

D LFC and Scale Sensitivity Analyses of a Real Microbiome Dataset

In Section *LFC Sensitivity Analysis and Testing of Real Data* and Fig 2 in the main text we presented a reanalysis of a real RNA-seq experiment using LFC Sensitivity Analysis. Here we present a reanalysis of a real 16S rRNA-seq experiment using LFC Sensitivity Analysis and real microbe sets. These results show that the findings presented in the main text for gene set enrichment analysis may be extended to a microbe

set enrichment analysis. We also present a Scale Sensitivity Analysis of this data to extend the results of Section A to 16S rRNA-seq data. We find that using GSEA-LFC-S as opposed to GSEA-LFC does not change our conclusion: both methods are sensitive to errors in scale assumptions, and that this sensitivity is entity-set specific.

These sensitivity analyses were performed using data from the NYC Health and Nutrition Examination Study (NYC HANES). This study compared the oral microbiomes of smokers and non-smokers Section D.1). Three microbe sets were used corresponding to aerobic, anaerobic and facultative anaerobic microbes. In Fig D part a we show the results of an LFC Sensitivity Analysis performed on this dataset. In this LFC Sensitivity Analysis we measured how results changes as a function of scale error ($\epsilon^{\perp}$) ranging from $[-1, 1]$. We find, similar to the RNA-seq study, that different microbe sets demonstrate varying degrees of scale sensitivity. The aerobic microbe set was completely insensitive to scale error. The Faculative anaerobic (F. anaerobic) microbe set was similarly insensitive as it is only significant when scale assumption error $\epsilon^{\perp}$ happens to be exactly -0.05: at all other values of $\epsilon^{\perp}$ (including when the scale assumption is correct, i.e., $\epsilon^{\perp} = 0$) the F. anaerobic microbe set is not significantly enriched or depleted. However the anaerobic microbe set showed a much higher degree of sensitivity: it is not longer significant when $\epsilon^{\perp}$ is in the range $[-0.15, -0.6]$.

We see similar results for our Scale Sensitivity Analysis of GSEA-LFC-S (Fig D part b). For this analysis we performed a Scale Sensitivity Analysis similar to that presented in Section A (see Section D.1 for full methods). The results of this analysis are similar to those of the LFC Sensitivity Analysis presented in Fig D part a, except the F. anaerobic microbe set is now completely insensitive to error as it is always found insignificant.

Together the results of these two sensitivity analyses reaffirm that scale sensitivity is a concern not just for gene set enrichment analysis using real RNA-seq data but for enrichment analyses of microbe sets using real 16S rRNA-seq data as well.

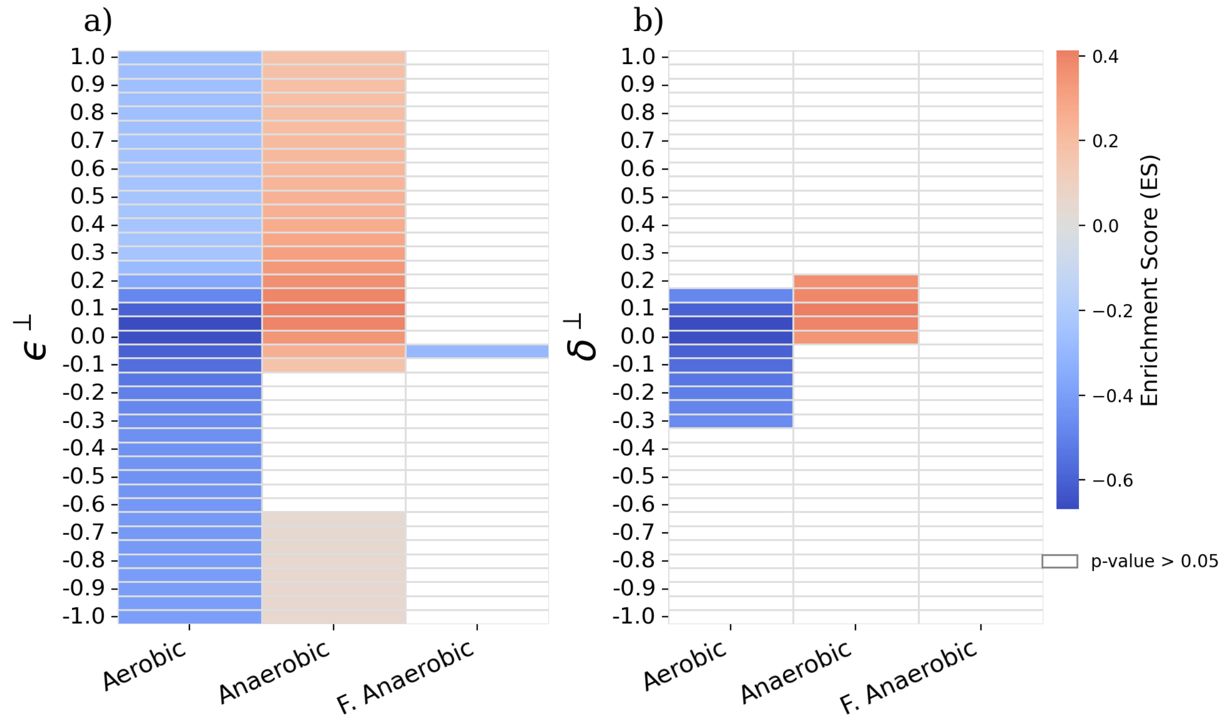

Figure D: Both GSEA-LFC and GSEA-LFC-S are sensitive to scale assumptions in the context of microbe set enrichment analysis. Here a 16S rRNA-seq data set comparing the oral microbiomes of smokers against non-smokers is used with three microbe sets. (a) LFC Sensitivity Analysis of GSEA-LFC. (b) Scale Sensitivity Analysis of GSEA-LFC-S. Errors $\epsilon^{\perp}$ and $\delta^{\perp}$ in the CLR scale assumption are shown on the Y-axis in both plots. Higher (or lower) values of $\epsilon^{\perp}$ or $\delta^{\perp}$ correspond to a higher (or lower) scale in smoker compared to non-smoker samples than assumed. Higher values (red) of the Enrichment Score (ES) indicate enrichment in smokers, while lower values (blue) correspond to enrichment in non-smokers.

D.1 Methods for 16S rRNA-seq LFC and Scale Sensitivity Analyses

Sensitivity analyses were performed using raw read counts from the NYC HANES 16S rRNA-seq oral microbiome dataset [13] comparing the oral microbiomes of non-smokers ($n = 43$) to smokers ($n = 86$) that were processed as described by Beghini et al. [14]. Three microbe sets representing aerobic, anaerobic, and facultative anaerobic microbes were used in the analysis [14]. For all analyses, LFCs were estimated after adding a pseudo-count of 0.1 to the CLR transformed read counts using Eq (5) in the main text. The error range $[-1, 1]$ was chosen for symmetry.

For the Scale Sensitivity Analysis, errors $\alpha_1, \ldots, \alpha_n$ were added to the CLR transformed read count matrix before sample-label permutations as

$$\alpha_n^{\perp} = \begin{cases} \delta^{\perp}, & \text{if } x_n = 1 \\ 0, & \text{if } x_n = 0. \end{cases}$$

Here $x_n = 1$ indicates smokers and $x_n = 0$ indicates non-smokers. After adding the error $\epsilon^{\perp}$ or $\delta^{\perp}$, p-values were calculated using GSEA [4] with 5000 sample-label permutations, which we refer to as GSEA-LFC-S.

E LFC Sensitivity Testing

We explored the statistical power of the LFC Sensitivity Test in the context of real data by applying it to the same thyroid tissue dataset analyzed in the Section *LFC Sensitivity Analysis and Testing of Real Data* of the main text. Calculating statistical power (i.e., the probability of detecting a true positive) typically requires simulation studies in order to know which gene sets are truly enriched or depleted. As we lack validated approaches for simulating datasets with known set enrichment we instead used LFC Sensitivity Analysis to calculate statistical power as a function of potential error in scale assumptions. We performed this analysis on the thyroid dataset introduced in the main text along with a set of 4441 gene sets (see methods in Section E.1). The results of this power analysis are shown in Fig E. We find that the average statistical power for the LFC Sensitivity Test is likely in the range of 6% to 13.5%. Out of the 4441 gene sets tested, the LFC Sensitivity Test identified 96 entity sets as significantly enriched or depleted. These results show that the LFC Sensitivity Test exhibits non-zero power and thus may have practical utility in research.

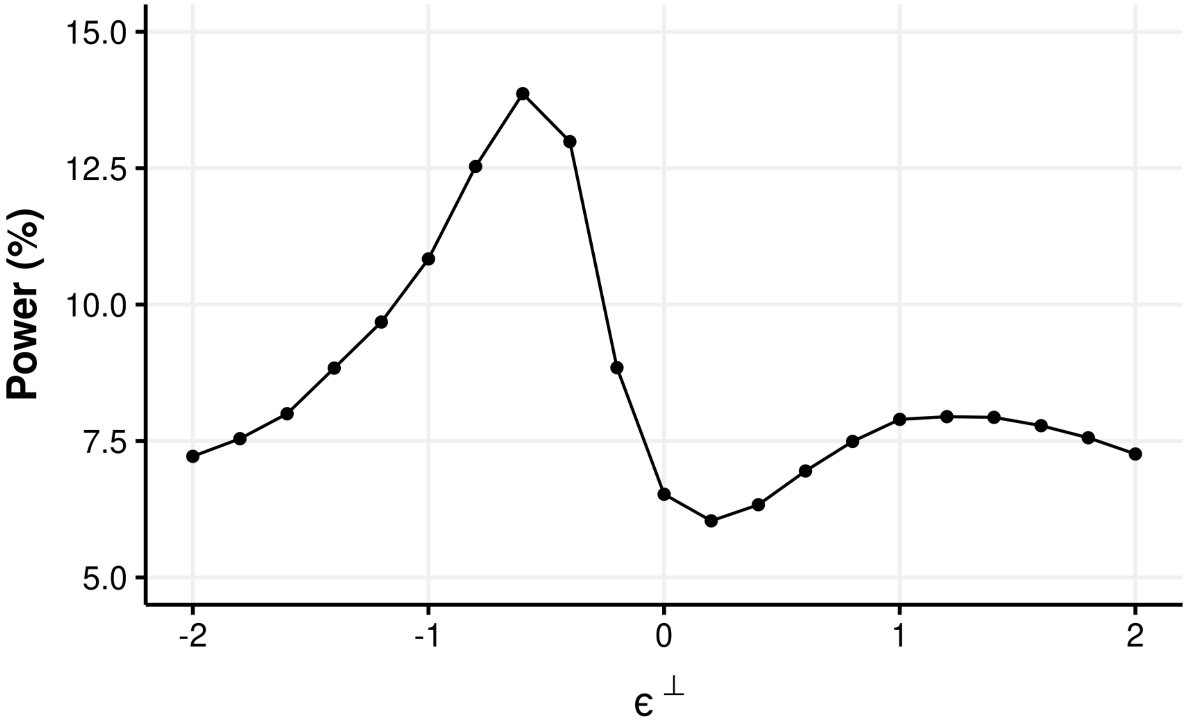

Figure E: The LFC Sensitivity Test has non-zero power. The thyroid normal-adjacent-to-tumor versus healthy tissue data set described in the Section *Preprocessing and Differential Analysis of Thyroid Tissue RNA-seq Data* in the main text was used along with 4441 gene sets to calculate power. Power is calculated as the percentage of gene sets identified as significant with GSEA-LFC when $\theta = \hat{\theta} + \epsilon^{\perp}$ that were also identified as significant with the LFC Sensitivity Test using a significance of $p < 0.05$.

E.1 Methods

Power was estimated using LFCs estimated from the thyroid normal-adjacent-to-tumor versus healthy study as described in the Section *Preprocessing and Differential Analysis of Thyroid Tissue RNA-seq Data* of the main text and using the MSigDB C2 (version 7.4.0) curated list of gene sets [4, 11]. Based on the default parameters of the GSEA software package [4], only gene sets that containing between 15 and 500 genes were retained in our analysis resulting in a set of 4441 candidate gene sets. Power is calculated at any single value of $\epsilon^{\perp}$ as the percentage of gene sets that GSEA-LFC identified as significant that LFC Sensitivity Testing (using GSEA-LFC) also identified as significant ($p < 0.05$). p-values for the LFC Sensitivity Test (e.g., $p = \max_{\epsilon^{\perp}} p_{\epsilon^{\perp}}$) were calculated over range of $\epsilon^{\perp} \in [-200, 200]$ with a grid size of 1, except in the range $\epsilon^{\perp} \in [-10, 10]$ where a grid size of 0.1 was used for better resolution. In all cases a significance threshold of $p < 0.05$ was used.

F GSEA with Compositional Weighting

This section consists of three parts. In the first part we prove that the LFC of an entity d (i.e., θ_d) used in GSEA-LFC is related to the GSEA-CLR input ϑ_d by

$$\vartheta_d = \theta_d - \mathrm{mean}(\theta).$$

In the second part we define GSEA with Compositional Weighting (GSEA-CW). We demonstrate that GSEA-CW is a generalization of GSEA-CLR and that GSEA-CW target estimands are scale invariant. In the final part we discuss another possible GSEA-CW target estimand and explore the scientific question it implies.

F.1 GSEA-CLR

Here we prove that ϑ_d (Eq (5) in the main text) is related to θ_d (Eq (3) in the main text) through the equality $\vartheta_d = \theta_d - \mathrm{mean}(\theta)$. We note that $W^{clr}_{dn} = \log[W_{dn}/G(W_{\cdot n})]$ where $G(W_{\cdot n})$ denotes the geometric mean of the vector $W_{\cdot n} = (W_{1n}, \ldots, W_{Dn})$. The system's scale (i.e., $W^{\perp}$) cancels out of the fraction $\log[W_{dn}/G(W_{\cdot n})]$, and thus W^{clr}_{dn} can be equivalently expressed entirely in terms of the system's composition (i.e., $W^{\parallel}$): $W^{clr}_{dn} = \log[W^{\parallel}_{dn}/G(W^{\parallel}_{\cdot n})]$. This can be shown mathematically as:

$$\begin{aligned}
W^{clr}_{dn} &= \log \frac{W_{dn}}{G(W_{\cdot n})} \\
&= \log \frac{W^{\parallel}_{dn} W^{\perp}_{n}}{\prod_{i=1}^{D} (W^{\parallel}_{in} W^{\perp}_{n})^{1/D}} \\
&= \log \frac{W^{\parallel}_{dn} W^{\perp}_{n}}{\prod_{i=1}^{D} (W^{\parallel}_{in})^{1/D} \prod_{i=1}^{D} (W^{\perp}_{n})^{1/D}} \\
&= \log \frac{W^{\parallel}_{dn} W^{\perp}_{n}}{\prod_{i=1}^{D} (W^{\parallel}_{in})^{1/D} W^{\perp}_{n}} \\
&= \log \frac{W^{\parallel}_{dn}}{\prod_{i=1}^{D} (W^{\parallel}_{in})^{1/D}} \\
&= \log \frac{W^{\parallel}_{dn}}{G(W^{\parallel}_{\cdot n})}
\end{aligned}$$

Expanding on Eq (4) in the main text, and using the the fact that $W^{clr}_{dn} = \log[W^{\|}_{dn}/G(W^{\|}_{\cdot n})]$:

$$\begin{aligned}
\vartheta_d &= \underset{n:x_n=1}{\text{mean}}(W^{clr}_{dn}) - \underset{n:x_n=0}{\text{mean}}(W^{clr}_{dn}) \\
&= \underset{n:x_n=1}{\text{mean}}\left[\log \frac{W^{\|}_{dn}}{G(W^{\|}_{\cdot n})}\right] - \underset{n:x_n=0}{\text{mean}}\left[\log \frac{W^{\|}_{dn}}{G(W^{\|}_{\cdot n})}\right] \\
&= \underset{n:x_n=1}{\text{mean}}\left[\log W^{\|}_{dn} - \log G(W^{\|}_{\cdot n})\right] - \underset{n:x_n=0}{\text{mean}}\left[\log W^{\|}_{dn} - \log G(W^{\|}_{\cdot n})\right] \\
&= \underset{n:x_n=1}{\text{mean}}\left[\log W^{\|}_{dn}\right] - \underset{n:x_{n=1}}{\text{mean}}\left[\log G(W^{\|}_{\cdot n})\right] - \underset{n:x_n=0}{\text{mean}}\left[\log W^{\|}_{dn}\right] + \underset{n:x_{n=0}}{\text{mean}}\left[\log G(W^{\|}_{\cdot n})\right] \\
&= \left(\underset{n:x_n=1}{\text{mean}}\left[\log W^{\|}_{dn}\right] - \underset{n:x_n=0}{\text{mean}}\left[\log W^{\|}_{dn}\right]\right) + \underset{n:x_{n=0}}{\text{mean}}\left[\log G(W^{\|}_{\cdot n})\right] - \underset{n:x_{n=1}}{\text{mean}}\left[\log G(W^{\|}_{\cdot n})\right] \\
&= \theta^{\|}_d + \underset{n:x_n=0}{\text{mean}}\left[\log G(W^{\|}_{\cdot n})\right] - \underset{n:x_n=1}{\text{mean}}\left[\log G(W^{\|}_{\cdot n})\right] \\
&= \theta^{\|}_d + \underset{n:x_n=0}{\text{mean}}\left[\underset{j=1,\dots,D}{\text{mean}}\left(\log W^{\|}_{jn}\right)\right] - \underset{n:x_n=1}{\text{mean}}\left[\underset{j=1,\dots,D}{\text{mean}}\left(\log W^{\|}_{jn}\right)\right] \\
&= \theta^{\|}_d + \underset{j=1,\dots,D}{\text{mean}}\left[\underset{n:x_n=0}{\text{mean}}\left(\log W^{\|}_{jn}\right)\right] - \underset{j=1,\dots,D}{\text{mean}}\left[\underset{n:x_n=1}{\text{mean}}\left(\log W^{\|}_{jn}\right)\right] \\
&= \theta^{\|}_d + \underset{j=1,\dots,D}{\text{mean}}\left[\underset{n:x_n=0}{\text{mean}}\left(\log W^{\|}_{jn}\right) - \underset{n:x_n=1}{\text{mean}}\left(\log W^{\|}_{jn}\right)\right] \\
&= \theta^{\|}_d + \text{mean}\left(-\theta^{\|}\right) \\
&= \theta^{\|}_d - \text{mean}\left(\theta^{\|}\right).
\end{aligned}$$

To complete the proof we show that $\vartheta_d = \theta_d - \text{mean}(\theta)$ is equivalent to $\vartheta_d = \theta^{\|}_d - \text{mean}\left(\theta^{\|}\right)$ because of the equality $\theta_d - \text{mean}(\theta) = \theta^{\|}_d - \text{mean}\left(\theta^{\|}\right)$:

$$\begin{aligned}
\theta_d - \text{mean}(\theta) &= \left(\theta^{\|}_d + \theta^{\perp}\right) - \text{mean}\left(\theta^{\|} + \theta^{\perp}\right) \\
&= \theta^{\|}_d + \theta^{\perp} - \text{mean}(\theta^{\|}) - \theta^{\perp} \\
&= \theta^{\|}_d - \text{mean}(\theta^{\|}).
\end{aligned}$$

Thus $\vartheta_d = \theta_d - \text{mean}(\theta)$. Notably, because the scale term $\theta^{\perp}$ cancels out, ϑ_d may be calculated as $\theta_d - \text{mean}(\theta)$ from any estimated $\hat{\theta}$, regardless of the assumed value $\hat{\theta}^{\perp}$.

F.2 GSEA-CW as a Function of a Log-contrast of θ

Following our notation in the main text, let θ denote a vector of LFCs, $\theta^{\|}$ denote the corresponding vector of log-fold-changes in proportions, and $\theta^{\perp}$ be a scalar denoting the log change in the scale of the system between two experimental conditions. We can then write $\theta = \theta^{\|} + \mathbf{1}\theta^{\perp}$ where $\theta^{\|}$ is a vector of D elements as defined in the main text and $\mathbf{1}$ is a vector with every element equal to 1.

A log-contrast of θ is a multivariate function $l : \Theta \to \mathbb{R}^P$ defined as

$$l(\theta) = \Psi\theta$$

for a $P \times D$ contrast matrix Ψ satisfying the constraint

$$\sum_d \Psi_{pd} = 0 \quad \forall p \in \{1, \dots, P\}.$$

Note that log-contrasts are scale-invariant functions which we illustrate by showing that $l(\theta) = l(\theta^{\parallel})$:

$$\begin{aligned} l(\theta) &= \Psi\theta \\ &= \Psi\theta^{\parallel} + \Psi\mathbf{1}\theta^{\perp} \\ &= \Psi\theta^{\parallel} \\ &= l(\theta^{\parallel}) \end{aligned}$$

where we have used the fact that $\Psi\mathbf{1}\theta^{\perp} = 0$ which follows from the sum-to-zero constraint on the rows of Ψ. It follows that any real-valued function f of a log-contrast, i.e., $f(l(\theta))$ is also scale invariant as $f(l(\theta)) = f(l(\theta^{\parallel}))$.

We define the GSEA-CW(-S) (where the -S suffix implies sample-label rather than entity-label permutations) target estimand as GSEA using a ranking statistic of the form $R = f(l(\theta))$. Using the notation in the main text, the target estimand for GSEA-CW(-S) can be expressed as $\varphi_S = u(f(l(\theta)))$. To ensure scale invariance, we require that the method chosen to estimate LFCs (θ) does not rely on the scale for estimation of $\theta^{\parallel}$. This requirement is included to avoid trivial scale reliance being introduced into GSEA-CW(-S). Together this is sufficient to prove that the GSEA-CW(-S) target estimand is scale invariant.

The GSEA-CLR(-S) target estimand has the required form for GSEA-CW(-S), which can be proven by constructing the matrix Ψ as

$$\Psi_{pd} = \begin{cases} \frac{(N-1)}{N}, & \text{if } p = d \\ -\frac{1}{N}, & \text{otherwise.} \end{cases} \tag{1}$$

The D length vector of GSEA-CLR(-S) inputs may then be expressed as $\vartheta = \Psi\theta$ and the GSEA-CLR(-S) target estimand is therefore $\varphi_S = u(\vartheta)$.

F.3 Another GSEA-CW Target Estimand

There exists infinitely many GSEA-CW target estimands and each implies a different scientific question. Here we explore one such target estimand: GSEA applied to the *absolute value* of the log-fold-change in CLR normalized abundances. This target estimand may be expressed as $\varphi_S = u(\lambda)$ where the elements of λ

are defined as:

$$\lambda_d = \left| \theta_d - \frac{1}{D} \sum_{i=1}^{D} \theta_i \right|.$$

The log-contrast for λ can be expressed using the same matrix Ψ as GSEA-CLR but with an absolute sign added: $\lambda = |\Psi\theta|$.

The scientific question implied by this target estimand differs from GSEA-CLR in one critical way. Consider an entity set for which half of the entities are highly depleted and half the entities are highly enriched relative to the mean LFC. For GSEA-CLR, half these entities would cluster at the beginning of the ranked list and half would cluster at the end. For this target estimand, however, the absolute value allows all the entities in the set to cluster together at the beginning of the ranked list. In this example, GSEA-CW(-S) using this ranking statistic would likely result in a lower p-value and higher ES compared to GSEA-CLR(-S). This target estimand therefore implies that we are interested not only in entity sets that are exclusively enriched or depleted relative to the mean, but also in entity sets where a portion of the entities are enriched and the other portion are depleted relative to the mean.

G Balances

In Section F we defined a class of scale invariant target estimands based on GSEA [4] using log-contrasts. However, alternative scale invariant methods for DSA not based on GSEA have been recently proposed. Nguyen et al. [15] propose a scale invariant method for DSA based on the *balance* between entities in versus not in some set of interest. For each gene-set S, their balance has the form

$$f_S(W_{\cdot n}) \propto \log \frac{\mathrm{G}(W_{(d \in S)n})}{\mathrm{G}(W_{(d \notin S)n})}. \tag{2}$$

Their DSA method then evaluates a null hypothesis using entity-label permutations to test if $f_S(W_{\cdot n})$ is positive or negative value within each participant n for each set S.

At face value, this balance formulation is appealing: it is scale invariant and intuitive, equating differential enrichment or depletion to changes in the average abundance between set and non-set entities. However, as we demonstrate below it can be highly sensitive to outliers as might occur when an entity is incorrectly annotated and thus erroneously included in a set to which it does not belong.

G.1 Sensitivity of Balances and GSEA-CLR to Outliers

Here we illustrate the sensitivity of balances by considering measurements of 11 entities from two participants. We define a matrix of abundances W as

$$W = \begin{bmatrix} 308 & 305 & 301 & 119 & 107 & 107 & 103 & 100 & 96 & 92 & 4 \\ 5 & 5 & 5 & 5 & 5 & 5 & 5 & 5 & 5 & 5 & 5 \end{bmatrix}^T.$$

Let each of the two columns of W (notice the transpose) represent individual study participants. Let the indices of the entities in the set of interest be given by $S = \{1, 2, 3, 11\}$, meaning that the counts associated with S for the first participant are $(308, 305, 301, 4)$. Suppose that entity 11 is an annotation error and is not actually in the biological set of interest S. In the first participant, the first three entities in S have substantially enriched counts compared to the last entity which represents an outlier with a count of only 4. In the second participant, all entities have a count of 5. Yet the balance for both participants are approximately zero implying that S is not enriched in either participant. Our conclusions is that balances may not capture true variation in the presence of an outlier (e.g., annotation error). GSEA-CLR is however able to capture the difference between participants. Calculating the LFC as $\theta_d = \log(W_{d1}/W_{d2})$ for each entity, then calculating $\vartheta_d = \theta_d - \text{mean}(\theta_1, \ldots, \theta_D)$, and then running GSEA, we compute that the GSEA-CLR enrichment score is 0.98 which is close to the maximum score of 1, indicating a high degree of enrichment.

If the outlier (annotation error) is removed from the set (i.e., letting $S = \{1, 2, 3\}$) then GSEA-CLR estimate is largely unchanged: the enrichment score increases only slightly to 1. In contrast, when the outlier is removed from S the results implied by balances change substantially: for the first participant the balance increases to 1.5 while the balance for the second participant remains at 0. With this removal of the outlier, the balances now distinguish between the participants.

This example illustrates how balances are highly sensitive to outliers (e.g., annotation errors) while GSEA-CLR is comparably less sensitive.

References

[1] Gatti DM, Barry WT, Nobel AB, Rusyn I, Wright FA. Heading Down the Wrong Pathway: on the Influence of Correlation within Gene Sets. BMC Genomics. 2010;11(1):574. doi:10.1186/1471-2164-11-574.

[2] Wu D, Smyth GK. Camera: a competitive gene set test accounting for inter-gene correlation. Nucleic Acids Res. 2012;40(17):e133. doi:10.1093/nar/gks461.

[3] Tamayo P, Steinhardt G, Liberzon A, Mesirov JP. The limitations of simple gene set enrichment analysis assuming gene independence. Stat Methods Med Res. 2016;25(1):472–487. doi:10.1177/0962280212460441.

[4] Subramanian A, Tamayo P, Mootha VK, Mukherjee S, Ebert BL, Gillette MA, et al. Gene set enrichment analysis: A knowledge-based approach for interpreting genome-wide expression profiles. Proc Natl Acad Sci U S A. 2005;102(43):15545–15550. doi:10.1073/pnas.0506580102.

[5] Hung JH, Whitfield TW, Yang TH, Hu Z, Weng Z, DeLisi C. Identification of functional modules that correlate with phenotypic difference: the influence of network topology. Genome Biol. 2010;11(2):R23. doi:10.1186/gb-2010-11-2-r23.

[6] Aran D, Camarda R, Odegaard J, Paik H, Oskotsky B, Krings G, et al. Comprehensive analysis of normal adjacent to tumor transcriptomes. Nat Commun. 2017;8(1):1077. doi:10.1038/s41467-017-01027-z.

[7] Nixon MP, Letourneau J, David LA, Lazar NA, Mukherjee S, Silverman JD. Scale Reliant Inference; 2023.

[8] Li WX, He K, Tang L, Dai SX, Li GH, Lv WW, et al. Comprehensive tissue-specific gene set enrichment analysis and transcription factor analysis of breast cancer by integrating 14 gene expression datasets. Oncotarget. 2017;8(4):6775–6786. doi:https://doi.org/10.18632/oncotarget.14286.

[9] Lonsdale J, Thomas J, Salvatore M, Phillips R, Lo E, Shad S, et al. The Genotype-Tissue Expression (GTEx) project. Nat Genet. 2013;45(6):580–585. doi:10.1038/ng.2653.

[10] Rahman M, Jackson LK, Johnson WE, Li DY, Bild AH, Piccolo SR. Alternative preprocessing of RNA-Sequencing data in The Cancer Genome Atlas leads to improved analysis results. Bioinformatics. 2015;31(22):3666–3672. doi:10.1093/bioinformatics/btv377.

[11] Liberzon A, Subramanian A, Pinchback R, Thorvaldsdóttir H, Tamayo P, Mesirov JP. Molecular signatures database (MSigDB) 3.0. Bioinformatics. 2011;27(12):1739–1740. doi:10.1093/bioinformatics/btr260.

[12] Ritchie ME, Phipson B, Wu D, Hu Y, Law CW, Shi W, et al. limma powers differential expression analyses for RNA-sequencing and microarray studies. Nucleic Acids Research. 2015;43(7):e47–e47. doi:10.1093/nar/gkv007.

[13] Thorpe LE, Greene C, Freeman A, Snell E, Rodriguez-Lopez JS, Frankel M, et al. Rationale, design and respondent characteristics of the 2013–2014 New York City Health and Nutrition Examination Survey (NYC HANES 2013–2014). Prev Med Rep. 2015;2:580–585. doi:https://doi.org/10.1016/j.pmedr.2015.06.019.

[14] Beghini F, Renson A, Zolnik CP, Geistlinger L, Usyk M, Moody TU, et al. Tobacco exposure associated with oral microbiota oxygen utilization in the New York City Health and Nutrition Examination Study. Ann Epidemiol. 2019;34:18–25.e3. doi:https://doi.org/10.1016/j.annepidem.2019.03.005.

[15] Nguyen QP, Hoen AG, Frost HR. CBEA: Competitive balances for taxonomic enrichment analysis. PLoS Comput Biol. 2022;18(5):1–24. doi:10.1371/journal.pcbi.1010091.
