# Supplementary material for: Addressing erroneous scale assumptions in microbe and gene set enrichment analysis
Source: PLoS Comput Biol. 2023 Nov 20;19(11):e1011659. doi: 10.1371/journal.pcbi.1011659 (PMC10695402; doi:10.1371/journal.pcbi.1011659)
Supplement: Attachment — Submitted filename: response.pdf [file pcbi.1011659.s002.pdf]

We appreciate the reviewers' thoughtful and helpful comments. In response, we made numerous changes throughout the manuscript which we believe enhance the clarity and accuracy of our presentation. Below we respond point-by-point to reviewer comments. Line numbers refer to positions within the revised manuscript.

Reviewer 1

As the authors stated in the manuscript, they are proposing a sensitivity measurement and a statistical test for controlling false positive rates of differential set analysis, potentially due to technical factors such as limitations of the sequence counting measurements. However, the contents of the manuscript should be reorganized to clearly distinguish their own contributions and the novelty from the previous results. This makes the paper difficult to read although the problem considered in this work mathematically sounds and could be attractive to biomedical research.

We agree with the reviewer that the separation between previous results and our contributions was unclear. We have modified the language we use throughout the introduction to better emphasize the distinction between prior work and our contributions. For example, we now clearly delineate where we switch from discussing prior work to our own contributions at the end of our review:

> To date, SRI has primarily been used to study target estimands associated with differential analysis. In what follows, we provide the first application of SRI to DSA.
> (Lines 146-147)

In addition, we have made numerous grammatical changes to emphasize the distinction. For example, we have changed language in our review of SRI from "We define X as" to "X is defined as" or "Nixon et al. define X as".

I think the approach could be described better, first at a high-level and then also formally.

We agree with the reviewer. We have made two changes which we think should improve the clarity of our methods. First we have added a new section entitled *Conceptual Overview of Methods* (Lines 149-165). Just as the reviewer suggests, this new (brief) section provides a high-level overview of the methods introduced throughout this work. Second, we have consolidated our introduction of methods in to a single section called *LFC Sensitivity Analysis and Testing*. Notably this improves readability compared to the prior draft where we only introduced LFC Sensitivity Testing at the end of a section that had been devoted to applying LFC Sensitivity Analysis to real data.

In the application to real data, what is conclusion related to biological knowledge?

The reviewer brings up a good point: our initial submission did not clearly state the biological implications of our work. Overall, our work argues for caution: that scale sensitivity can be substantial in the context of real data and that conclusions that are sensitive to such errors should be viewed skeptically. To address this comment we have added the following paragraph to our discussion:

> Our results suggest caution is warranted when performing DSA from sequence count data as we do find that results can be highly sensitive to even slight errors in scale assumptions. For example, in the reanalysis of Aran et al. [1] we found that gene sets in the Apoptosis and Interferon Alpha Response pathways, which were reported by those authors as enriched (in Thyroid normal-adjacent-to-tumor tissue), were no longer significant with scale assumption error as small as $\epsilon^{\perp} = -0.05$. To be clear, we are not arguing that those conclusions are wrong: we do not know the ground truth LFCs. Instead, we have only studied the sensitivity of those conclusions to errors in modeling

assumptions. Still, we find such sensitivity concerning and suggest that such conclusions should be viewed skeptically.
(Lines 382-391)

Reviewer 2

This is a well written and reproducible article addressing an important practical problem from a rather theoretical perspective while providing practical solutions.

We thank the reviewer for their support.

As a general comment, I would say that the mathematical notations are quite hard to follow (even for someone with a mathematical background). It may be useful to include a table with an overview of these notations with corresponding definitions.

We agree with the reviewer's comment and have added a table of notation and corresponding definitions starting at line 75. In addition, we have simplified our notation. For example, we no longer use the tilde ($\tilde{\cdot}$) notation which was non-standard.

Regarding the structure of the manuscript, I understand that the proposed methods are actually the results of this methodological article. However, I find the structure of the paper a bit confusing, since the Results section contains both the description of methods and numerical results. A structure with the (new) methods before the results would appear more appropriate - if allowed by the journal's policy.

We agree with the reviewer, the structure of methods and results in our prior draft was confusing. We believe this confusion stemmed from our introduction of LFC Sensitivity Testing at the end of a section that was dedicated to illustrating the performance of LFC Sensitivity Analysis on real data. This has been fixed. In our updated manuscript we now introduce all new methods in a section called *LFC Sensitivity Analysis and Testing*. With this change subsequent results sections now focus solely on applying these methods (sections titled "The GSEA-LFC Target Estimand is Typically Scale Reliant" and "LFC Sensitivity Analysis and Testing of Real Data") or discussing how the scientific question dictates the impacts of erroneous scale assumptions (section titled "GSEA with Compositional Weighting").

Please define the term "compositional survey" the first time it occurs.

We thank the reviewer for pointing out this oversight. In fact, we only used this term once throughout the initial submission, in our revised submission we have simply removed the term as the definition of compositional surveys is not essential to our presentation. What we had called a "compositional survey" in the initial submission is now simply referred to as "observed data".

"A unique estimate of $\hat{\psi}$": the authors probably mean ψ rather than $\hat{\psi}$?

The reviewer is correct. This has been fixed.

It is not clear to me why Eq.(5) results from a simplification of the assumption. Please explain.

This comment was also mentioned by Reviewer 3 and we agree that this was not clear. In our revised manuscript we have expanded our presentation and now provide a more comprehensive step-by-step explanation of how Equation 5 is derived. In addition, we have added a supplementary section (Supplementary Section 6) which provides an even more detailed derivation of the equation. Note that in the revised manuscript what had been Equation 5 is now is now Equation 6. In addition, we

have fixed a mistake in that equation which we expect also contributed to the reviewers' confusion (what had been written $\vartheta^{\perp}$ should actually have been $\theta^{\perp}$). Below we include the section of the revised manuscript for the reviewers convenience. The revised section can be found between Lines 128 and 145.

> In brief, these methods [methods like ALDEx2 or Songbird that calculate LFCs from CLR normalized abundances] can be thought of as producing estimates of a target estimand:
>
> $$\vartheta_d = \underset{n:x_n=1}{\text{mean}}(\log W^{clr}_{dn}) - \underset{n:x_n=0}{\text{mean}}(\log W^{clr}_{dn}) \tag{4}$$
>
> where $W^{clr}_{dn} = \log[W_{dn}/G(W_{\cdot n})]$ and where $G(W_{\cdot n})$ denotes the geometric mean of the vector $W_{\cdot n} = (W_{1n}, \dots, W_{Dn})$. The system's scale (i.e., $W^{\perp}$) cancels out of the fraction $\log[W_{dn}/G(W_{\cdot n})]$ (see Supplementary Section 6), and thus W^{clr}_{dn} can be equivalently expressed entirely in terms of the system's composition (i.e., $W^{\parallel}$): $W^{clr}_{dn} = \log[W^{\parallel}_{dn}/G(W^{\parallel}_{\cdot n})]$. The CLR target estimand may be further broken down into its compositional and scale components, similar to Eq 3), as
>
> $$\begin{aligned}\vartheta_d = &\underbrace{\left[\underset{n:x_n=1}{\text{mean}}\left(\log W^{\parallel}_{dn}\right) - \underset{n:x_n=0}{\text{mean}}\left(\log W^{\parallel}_{dn}\right)\right]}_{\vartheta^{\parallel}_d} + \\ &\underbrace{\left[\underset{n:x_n=1}{\text{mean}}\left(\log \frac{1}{G(W^{\parallel}_{\cdot n})}\right) - \underset{n:x_n=0}{\text{mean}}\left(\log \frac{1}{G(W^{\parallel}_{\cdot n})}\right)\right]}_{\vartheta^{\perp}} \\ = &\ \vartheta^{\parallel}_d + \vartheta^{\perp}.\end{aligned} \tag{1}$$
>
> The CLR target estimand in Eq (4) is not the same as the LFC target estimand in Eq (2). Yet many authors take the estimates produced by ALDEx2 and Songbird as estimates of the LFC as defined in Eq (2) (e.g., [2, 3]). While $\theta^{\parallel} = \vartheta^{\parallel}$, $\theta^{\perp}$ (Eq 3) differs from $\vartheta^{\perp}$ (Eq 1) as the former is a function of the system's scale (i.e., $W^{\perp}$) and the latter is a function of the system's composition (i.e., $W^{\parallel}$). Assuming that the output of ALDEx2 or Songbird represents estimates of LFCs (as defined in Eq 2) is equivalent to an implicit assumption that $\theta_d = \vartheta_d$. This assumption can be further simplified to $\theta^{\perp} = \vartheta^{\perp}$ which is an assumption that the true log fold change in scale can be imputed from the system composition:
>
> $$\theta^{\perp} = \underset{n:x_n=1}{\text{mean}}\left(\log \frac{1}{G(W^{\parallel}_{\cdot n})}\right) - \underset{n:x_n=0}{\text{mean}}\left(\log \frac{1}{G(W^{\parallel}_{\cdot n})}\right). \tag{6}$$
>
> We refer to this as the CLR assumption.

On p6 it is stated "GSEA-LFC uses GSEA to test whether the LFCs of entities in the set cluster together non-randomly when compared to the LFCs of entities not in the set", suggesting that the authors consider competitive hyotheses. They could mention that this deviates from standard GSEA, that considers self-contained hypotheses.

We agree that additional clarity is warranted. In fact, the initial submission did not even need to discuss GSEA in this section which was instead focused on introducing LFC Sensitivity Analysis and Testing. To clarify, LFC Sensitivity Analysis and Testing can be applied to any two-step estimator

where the first step estimates LFCs and the second step uses the estimates of LFCs for some other estimation tasks. GSEA applied to LFCs is just one example of this form of two-step estimator. In our revised manuscript we have removed the discussion of GSEA-LFC and instead introduce it in the section titled "The GSEA-LFC Target Estimand is Typically Scale Reliant". In that later section we have also expanded out discussion to highlight that we consider both the competitive hypotheses and self-contained hypothesis formulations of GSEA. Note, we do not refer to them as such in this manuscript but instead refer to them as GSEA with entity label permutations or GSEA with sample label permutations as we feel the latter description requires less prior knowledge of the GSEA literature and is more relevant to the discussions in this article.

> There are two common variations on GSEA that differ in the permutation scheme used to test the significance of the estimated $\hat{\phi}_S$ statistic. The first permutes the entity labels. The second permutes the sample labels. Assuming adequate sample size, sample-label permutations are generally preferred, as they ensure that the null model accounts for inter-entity correlations [26-28]. Coupled with these two variants on GSEA, we can define two target estimands each based on applying GSEA to the true LFCs. We denote the estimand formed from GSEA with entity label permutations as GSEA-LFC and the estimand formed from GSEA with sample label permutations as GSEA-LFC-S. In the main text we focus on GSEA-LFC as sensitivity analyses with GSEA-LFC-S are more complex yet show similar results. Still, we present a form of sensitivity analysis for GSEA-LFC-S as well as other DSA methods such as CAMERA [27] in Supplementary Section 1.
> (Lines 238-249)

"We assume the target estimand and estimator are given by" - target estimand and estimator cannot be given by the same expression.

The reviewer is correct that our terminology did not fit with statistical conventions. We have removed the term target estimator from the revised manuscript as we only needed the term target estimand. That is, in the new manuscript we now have a target estimand (the quantity we wish to estimate) and an applied estimator (an estimator an analyst uses to estimate the target estimand).

Also, I do not understand why we need tilde to denote the true value. This does not fit usual conventions in statistics.

The reviewer is correct, this was unnecessary. We have removed this notation.

I do not understand the rationale of the assumption $\epsilon \approx \epsilon^{\perp}$.

We agree with the reviewer that our explanation of this point was not clearly articulated in our initial submission. In brief, our rational can be simply stated as: we make this assumption as our manuscript is focused on studying errors in scale assumptions. While there are certainly errors in compositional estimates as well, those later errors would only serve to complicate our study and are unlikely to change the major conclusions of our work. Consider that if a conclusion is highly sensitive to errors in scale assumptions, it is likely still sensitive to those errors regardless of whether or not there are additional errors in compositional estimates. Our revised manuscript now provided an expanded explanation of this point:

> While there can be error in compositional estimation such that, for some d, $\epsilon^{\parallel}_d \neq 0$, the consideration of such error only serves to complicate our study of scale. Consider

that unless $\epsilon^{\perp}$ and $\epsilon_d^{\parallel}$ are strongly anti-correlated, if a conclusion is highly sensitive to error $\epsilon^{\perp}$ when $\epsilon_d^{\parallel} = 0$, it will still be highly sensitive when $\epsilon_d^{\parallel} \neq 0$. Thus, we restrict our analysis to $\epsilon^{\perp}$ by assuming that $\epsilon_d^{\parallel} \ll \epsilon^{\perp}$ such that $\epsilon \approx \mathbf{1}\epsilon^{\perp}$.
(Lines 190-194)

Please check the references carefully. Some of them are incomplete/incorrect (esp. article numbers of online journals).

We thank the reviewer for pointing out these errors. We have fixed them in the references section of the updated manuscript.

Reviewer 3

In the abstract the headline figure for false positive rates is 70%, but I'm not sure what part of the text this is referring to? Is it the analysis of simulated data?

The reviewer is correct this was not clear. We have updated the introduction to more clearly align with results explicitly discussed in the main text. The relevant sentence of the abstract has been updated to read:

> We show that even small errors in these assumptions can lead to positive predictive values as low as 9%.

This number aligns with the results shown in figure 1c. The main text now reflects this:

> Fig 1c summarizes those results and shows that the PPV can decrease to below 50% with errors on the order of ± 0.5. In words, when the average difference in scales between the two conditions is 1.65 times larger ($e^{0.5}$) or 0.6 times lower ($e^{-0.5}$) than assumed, more than half of the entity sets identified as significantly enriched or depleted are false positives. At $\epsilon = 1$ the PPV drops to just 9%.
> (Lines 260-264)

The "CLR assumption" is used in the analysis of real data and simulated data to demonstrate the main point of the paper, so I think it would be helpful to cite other work where this assumption has been used.

This is a good idea. Our revised manuscript now explicitly cites examples of prior work that has used this assumption:

> Yet many authors take the estimates produced by ALDEx2 and Songbird as estimates of the LFC as defined in Eq (2) (e.g., [2, 3]).
> (Lines 137-138)

For the reviewers convenience, we include references to those two citations at the end of this document.

The derivation of equation (5) is not clear to me. It would be helpful if this could be added (possible to the supplementary materials), and a description of the proportional and scale components of v added to the text.

We thank the reviewer for pointing this out. This comment was also made by Reviewer 2. In brief, we have responded to this comment by providing an expanded derivation of Equation 5 in the main text, adding a description of the proportional and scale components of ϑ, and also adding

a supplemental section that provides an even more detailed derivation than that added to the main text. For further details on these changes, please see our response to Reviewer 2 above.

It would be helpful to have a description of what the plausible error is in terms of the experiment parameters for section 2.4. There is some discussion of this in section 4.2 but I think it would be helpful to give an example in 2.4, i.e. what does a scale error of -0.1 translate as in terms of the experimental data?

We agree with the reviewer that additional context for what a scale error of $\epsilon = -0.1$ means would improve clarity. We have therefore added the following explanation on how to more easily interpret scale error:

> With an error as small as $\epsilon^{\perp} = -0.05$ multiple gene sets identified as enriched would no longer be significant (e.g., the columns Inflammatory Response to DNA Repair). To interpret the magnitude of this error, consider that for this dataset the CLR assumption equates to $\hat{\theta}^{\perp} = -0.3$ implying that if the mean scale in the normal-adjacent-to-tumor tissue is at least $\exp(-0.3 - 0.05) = 0.70$ times lower than the healthy tissue, then multiple significant gene sets are false positives.
> (Lines 286-292)

The discussion says that "sensitivity analyses provide a powerful tool for mitigating these problems". I'm not sure that this has been demonstrated. Similarly, the next sentence says "this work provides tools to mitigate the impact of erroneous scale assumptions". Certainly it provides tools to identify them and therefore avoid them, or at least acknowledge them, but I'm not sure what the tools provided that mitigate them are. Perhaps this could be reworded to be a little clearer.

The reviewer is correct, we have not actually demonstrated that our tools can mitigate these problems as that would likely require a study of analyst interpretations of their modeling results before compared to after performing this form of sensitivity analysis. We have updated our language to be more correct. We no longer state that our tools can mitigate these problems, instead we simply state that our tools can help identify when conclusions regarding a particular entity set is highly sensitive to errors in scale assumptions.

> In all these cases, we arrived at the same three conclusions: 1. for common scientific goals, even slight errors in scale assumptions can lead to false positives in DSA; 2. sensitivity analyses enable researchers to identify those gene and microbe sets whose significance is highly sensitive to errors in scale assumptions; 3. the sensitivity of DSA to errors in scale assumptions depends on the goal of the study.
> (Lines 376-381)

There are several typos throughout the text, including places where math-mode is not used but I think it should be (e.g. for "p-value" and "n=92" in the caption for Figure S1). I leave a list of these for the copy editor.

These typos have been fixed.

There are several cases where the same paper is cited many times, even in the same paragraph. I think it is sufficient to give the citation only the first time that the paper is mentioned.

We have removed some of the more repetitive references.

The abstract states "Second, we introduce a statistical test that provably controls Type-I error at a nominal rate", yet in the text the exposition of the statistical test is relegated to the supplementary

materials. If this part of the abstract is supposed to mirror the outline of the paper then maybe it should say something like "Second, we demonstrate on real and simulated data the Type-I error that can occur with incorrect scale assumptions."?

We thank the reviewer for this comment. To address this concern we have moved much of the explanation of the LFC Sensitivity Test from the Supplement to the main text (Lines 214-228). As part of this change we have renamed the Results section "LFC Sensitivity Analysis" to "LFC Sensitivity Analysis and Testing". Similarly we now provide numerical results on the power of the LFC Sensitivity Test in the section now called "LFC Sensitivity Analysis and Testing" (Lines 293-299). With these changes the language of the abstract now aligns the paper.

In the first paragraph of Section 1.1.1 I think "DNA molecules" should be replaced with "DNA reads" or "DNA sequences".

Fixed: "DNA molecules" has been changed to "DNA sequences" in the revised manuscript.

In the first paragraph of Section 1.1.2, "...that produces a unique estimate of $\hat{\psi}$ must have..", I think the hat should be removed.

The reviewer is correct. This has been fixed in the revised manuscript.

I don't think the definition of W_{dn}^{clr} after equation (4) is consistent with its use in supplementary section 6.1. It seems like there are too many logs (i.e., one in equation (4) itself and one in the definition of W_{dn}^{clr}). Is this right?

We thank the reviewer for pointing out this error. The extra log in the supplement has been removed.

In section 1.1.2, the abbreviation CLR is used before it is defined.

This has been fixed. The first mention of the CLR abbreviation now reads:

> Following Nixon et al. [14], we illustrate the concept of scale assumptions through a study of Centered Log-Ratio (CLR) normalization in differential abundance analysis.
> (Lines 125-126)

In the first paragraph of Section 2.3 ,"The GSEA algorithm is visualised in Figure 1..." – I don't think it is. Is this referencing a figure that is no longer present?

We thank the reviewer for this comment as it revealed a lack of clarity in our original statement. In the originally submitted manuscript we did not explain how Figure 1a and Figure 1b help visualize the GSEA algorithm. In the revised manuscript we now explain how the figure illustrates key steps of the GSEA algorithm:

> The GSEA algorithm is visualized in Fig 1 and a formal definition is provided in Supplementary Section 2. In brief, GSEA is an algorithm that first orders entities by a ranking statistic (here LFCs; Fig 1a), then measures the degree of non-random clustering in the ranked list through an enrichment score calculated as the maximum distance of a weighted running sum (Fig 1b), and then performs a permutation test to determine whether that enrichment score is unusually large or small.
> (Lines 231-236)

I found the arrows and text in Figure 3 a little bit confusing. I understand what they are trying to demonstrate but I'm not sure that it is correct.

We think this confusion may stem from a lack of appropriate indexes in the figure legend. This has been fixed. Regarding correctness, we have added a derivation of the relationships underlying this figure in Supplementary Section 6. Echoing these changes, we have added the following text to the legend of Figure 3:

> The index 1 refers to the leftmost X in the plot. mean$(\theta_1, \ldots, \theta_D)$ is the mean of distance of all X's and O's from 0 ... The equality $\vartheta_1 = \theta_1 - \text{mean}(\theta_1, \ldots, \theta_D)$ is derived in Supplementary Section 6.
> (Figure 3 legend, lines not numbered)

In Supplementary section 1, second equation, should $\alpha_n^{\perp}$ be 1 when $x_n = 0$? In the text following it looks like it should be 0.

We thank the reviewer for pointing out this error, it has been fixed.

In Supplementary section 4, the text says, "The facultative anaerobic (F. Anaerobic) microbe set, on the other hand, was highly sensitive as it was only enriched at a single value." I'm not sure that I agree with describing this as highly sensitive – surely it is the opposite?

We think this confusion is the result of two different possible perspectives on the sensitivity of an entity set. From the first perspective, which we took in the original manuscript, the f. anaerobic set is highly sensitive because a small change in $\epsilon^{\perp}$ from 0 to -0.05 changes its significance. From the reviewer's perspective it is highly insensitive because it is *only* significant under the condition that the true scale differs from the scale assumption by exactly $\epsilon^{\perp} = -0.05$. We agree with the reviewer's perspective and have changed the language accordingly:

> The aerobic microbe set was completely insensitive to scale error. The Faculative anaerobic (F. anaerobic) microbe set was similarly insensitive as it is only significant when scale assumption error $\epsilon^{\perp}$ happens to be exactly -0.05: at all other values of $\epsilon^{\perp}$ (including when the scale assumption is correct, i.e., $\epsilon^{\perp} = 0$) the F. anaerobic microbe set is not significantly enriched or depleted. However the anaerobic microbe set showed a much higher degree of sensitivity: it is not longer significant when $\epsilon^{\perp}$ is in the range $[-0.15, -0.6]$.
> (Supplement, Lines 147-152)

In Supplementary section 6.1 a detailed derivation is given (which is great). But the first step where $W_n^{\perp}$ cancels is not shown explicitly.

We have added a full derivation showing why $\log[W_{dn}/G(W_{\cdot n})] = \log[W_{dn}^{\parallel}/G(W_{\cdot n}^{\parallel})]$ in Supplementary Section 6:

$$
\begin{aligned}
W_{dn}^{clr} &= \log \frac{W_{dn}}{G(W_{\cdot n})} \\
&= \log \frac{W_{dn}^{\parallel} W_{n}^{\perp}}{\prod_{i=1}^{D} (W_{in}^{\parallel} W_{n}^{\perp})^{1/D}} \\
&= \log \frac{W_{dn}^{\parallel} W_{n}^{\perp}}{\prod_{i=1}^{D} (W_{in}^{\parallel})^{1/D} \prod_{i=1}^{D} (W_{n}^{\perp})^{1/D}} \\
&= \log \frac{W_{dn}^{\parallel} W_{n}^{\perp}}{\prod_{i=1}^{D} (W_{in}^{\parallel})^{1/D} W_{n}^{\perp}} \\
&= \log \frac{W_{dn}^{\parallel}}{\prod_{i=1}^{D} (W_{in}^{\parallel})^{1/D}} \\
&= \log \frac{W_{dn}^{\parallel}}{G(W_{\cdot n}^{\parallel})}
\end{aligned}
$$

(Supplement, Line Number 208)

I'm not sure what $n = [1, 2]$ means in the second paragraph of supplementary section 7.1.

We agree with the reviewer that this notation was unclear. It has been removed:

> Let each of the two columns of W (notice the transpose) represent individual study participants. Let the indices of the entities in the set of interest be given by $S = \{1, 2, 3, 11\}$, meaning that the counts associated with S for the first participant are $(308, 305, 301, 4)$. Suppose that entity 11 is an annotation error and is not actually in the biological set of interest S.
> (Supplement, Lines 265-268).

References

1. Aran, D. *et al.* Comprehensive analysis of normal adjacent to tumor transcriptomes. *Nat. Commun.* **8,** 1–13 (2017).
2. Chung, C. Z. *et al.* RNA surveillance by uridylation-dependent RNA decay in Schizosaccharomyces pombe. *Nucleic Acids Research* **47,** 3045–3057. `https://doi.org/10.1093/nar/gkz043` (Feb. 2019).
3. Gicquelais, R. E., Bohnert, A. S. B., Thomas, L. & Foxman, B. Opioid agonist and antagonist use and the gut microbiota: associations among people in addiction treatment. *Scientific Reports* **10.** `https://doi.org/10.1038/s41598-020-76570-9` (Nov. 2020).
